# Supplementary material for: Influencing Busy People in a Social Network
Source: PLoS One. 2016 Oct 6;11(10):e0162014. doi: 10.1371/journal.pone.0162014 (PMC5053606; doi:10.1371/journal.pone.0162014)
Supplement: S1 Appendix — (PDF) [file pone.0162014.s001.pdf]

# Influencing Busy People in a Social Network

Kaushik Sarkar[1*], Hari Sundaram[2*]

**1** School of Computing, Informatics and Decision Systems Engineering, Arizona State University, Tempe, Arizona, USA
**2** Dept. of Computer Science, University of Illinois at Urbana-Champaign, Urbana, Illinois, USA

* ksarkar1@asu.edu, hs1@illinois.edu

# Appendix

## A Computation of Immediate Adoption Probability

In this section we discuss an example of how the different immediate behavior adoption probabilities for a node are computed. This computation depends on whether all the thresholds of a node have the same random value (*matched threshold*) or independent and uniformly distributed random values (*different threshold*). Although all the results in this paper are for the *different threshold* model, here we present examples for both cases for the sake of completeness. Suppose a vertex $v$ has 8 neighbors. According to our threshold model each of its neighbors exerts an influence of 0.125 on it. Suppose $v$ is already a seed for behavior $A$; moreover it has 2 neighbors with behaviors $B$, and 3 neighbors with behavior $C$. We are interested in computing the probabilities that it will adopt each of the three behaviors in the next time step.

### A.1 Matched Threshold:

In this case, for any vertex the thresholds for all the three behaviors will be the same, but it will be assigned independently of other nodes and uniformly at random from the interval $[0, 1]$. So if $v$'s threshold is in the interval $[0, 0.25]$, then $v$ will consider both behaviors B and C together with A for adoption. Our payoff maximizing behavior adoption process dictates that it will adopt a subset of $A$,$B$ and $C$ that will provide maximum combined payoff subject to the resource constraint of the node. This adoption decision process is equivalent to solving a knapsack problem. We will solve the knapsack problem and decide which behaviors out of the three behaviors - A, B and C - will be adopted. Any such behavior will be adopted with probability 0.25.

If $v$'s threshold is in the interval $(0.25, 0.375]$ then $v$ will only consider behavior C together with behavior A for adoption. Again after solving knapsack problem and deciding which behaviors to adopt out of A and C, it will adopt any such behavior with probability 0.125.

At last if $v$'s threshold is in the interval $(0.375, 1]$ then it will definitely adopt behavior A - the probability of which is $1 - 0.375 = 0.625$. In the worst case the complexity of this probability computation process for each node is linear in the number of behaviors.

### A.2 Different Threshold:

In the *different threshold* case, for each vertex the thresholds are assigned independently and uniformly at random from the interval $[0, 1]$. So in this case we need to consider all possible combinations of behaviors B and C together with A (which will always be considered) and work out the individual probabilities. The worst case computational complexity of this process for each node will be exponential in the number of behaviors. In our example we need to consider the following cases:

i) B and C together with A; any behavior selected by the knapsack algorithm will be adopted with probability $0.25 \times 0.375 = 0.09375$.

ii) B together with A; any behavior selected by the knapsack algorithm will be adopted with probability $0.25 \times (1 - 0.375) = 0.15625$.

iii) C together with A; any behavior selected by the knapsack algorithm will be adopted with probability $(1 - 0.25) \times 0.375 = 0.28125$.

iv) Only A; A will be adopted with probability $(1 - 0.25) \times (1 - 0.375) = 0.46875$.

As this section illustrates, computation of this metric is considerably more complex and costly in comparison to node degree and influence weight based heuristics.

# B Variants of Seed Selection Algorithm

Table 1 presents the Total Participation and Total Adoption values for the different variants of the KKT seed selection algorithm and IA based seed selection heuristic. **T** — *topped up* versions provide better spread than the **NT** — *no top up* versions which is expected since more resource is required for starting the diffusion in the **T** version. However for the same type of top up regime there is not much difference between the **S** (*single* behavior per seed) and **M** (*multiple* behaviros per seed) version. If we consider exact algorithms instead of heuristics and approximation algorithms, then it is easy to see that **S** version can never produce a result that is better than the **M** version, since solution for **S** version is also a valid solution for **M** version. This fact accounts for the absence of any real difference between the **S** and **T** versions in the case of the heuristic and the approximate algorithm.

**Table 1.** Total Participation / Total Adoption under different networks as % of the network size. S and M variants give almost identical results with T variants exceeding NT variants.

| Heuristics | PA | SW | SC |
|---|---|---|---|
| KKT-S-T | 43.7 / 44.5 | 26.2 / 26.4 | 27.3 / 27.3 |
| H8-S-T | 43.9 / 44.5 | 23.6 / 24.5 | 23.6 / 24.2 |
| KKT-S-NT | 39.5 / 39.5 | 21.7 / 22.0 | 22.0 / 22.5 |
| H8-S-NT | 39.51 / 39.8 | 22.7 / 23.2 | 20.0 / 20.5 |
| KKT-M-T | 43.7 / 44.5 | 26.2 / 26.4 | 27.1 / 27.1 |
| H8-M-T | 39.0 / 45.8 | 22.8 / 23.5 | 21.9 / 22.6 |
| KKT-M-NT | 39.5 / 39.5 | 21.7 / 22.0 | 22.4 / 23.0 |
| H8-M-NT | 39.5 / 43.3 | 22.7 / 23.2 | 19.7 / 21.1 |

# C Equivalence between the Threshold and Network Average Cases

In table **??** we have seen that the resource utilization values under threshold and network average conditions are almost identical. In this section we will investigate the relationship between these two type of averages. First we will show an exact relation for the regular networks. This special case will provide us with helpful insights for analyzing the more general cases.

Suppose we have $n$ nodes with fixed resource distribution. Each node will have a fixed in-degree $\rho$. Each node selects $\rho$ in-neighbors uniformly at random from the rest $n-1$ nodes. We assume that only in-neighbors can exert influence on a node. In the *threshold average* (**TA**) case the nodes choose the in-neighbors at random at the beginning of the simulation and then at the start of each simulation run select the threshold values uniformly at random from the interval $[0,1]$. In the *network average* (**NA**) case each node chooses threshold values uniformly at random from the interval $[0,1]$ at the beginning of the simulation and then at the start of each simulation run it chooses its $\rho$ in-neighbors uniformly at random from the rest of the nodes. Both the processes start with a set $S$ of seeds for each of the $k$ behaviors. The diffusion process unfolds over time according to the Sticky multiple behavior diffusion process. We will show that $\sigma_{TA}(S) = \sigma_{NA}(S)$ by proving the following lemma:

*Lemma C.1.* For a given seed set $S$, the following two distributions over the sets of nodes are the same:

1. The distribution of probability over the active sets at the completion of the diffusion process in the **TA** case.
2. The distribution of probability over the active sets at the completion of the diffusion process in the **NA** case.

*Proof.* We prove the lemma by induction over the time step $t$. Clearly it is true at $t = 0$. Let $S_i^{(t)}$ denote the set of nodes with behavior $i$ at the end of time step $t$, and $S^{(t)} := \cup_i S_i^{(t)}$. For the **TA** case, suppose $v$ is a node that has not adopted any behavior at the end of time step $t$ and $\kappa(v) = \kappa \neq 0$. As before, the probability that $v$ will become active at the time step $t+1$, given that it was not active at the previous time step is -

$$\begin{aligned} &1 - \prod_{i=1}^{\kappa}\left(1 - \frac{\sum_{w \in S_i^{(t)} \setminus S_i^{(t-1)}} b_{v,w}}{1 - \sum_{w \in S_i^{(t-1)}} b_{v,w}}\right) \\ =&1 - \prod_{i=1}^{\kappa}\left(1 - \frac{\sum_{w \in S_i^{(t)} \setminus S_i^{(t-1)}} \frac{1}{\rho}}{1 - \sum_{w \in S_i^{(t-1)}} \frac{1}{\rho}}\right) \\ =&1 - \prod_{i=1}^{\kappa}\left(1 - \frac{|S_i^{(t)} \setminus S_i^{(t-1)}|}{\rho - |S_i^{(t-1)}|}\right) \end{aligned}$$

For the **NA** case, again let $v$ be a node that is not active at time step $t$ with $\kappa(v) = \kappa \neq 0$. The probability that $v$ will become active at time step $t+1$, given that it was not active till the previous time step is given by -

$$\begin{aligned} &1 - \prod_{i=1}^{\kappa}\left(1 - \frac{\sum_{w \in S_i^{(t)} \setminus S_i^{(t-1)}} b_{v,w}}{1 - \sum_{w \in S_i^{(t-1)}} b_{v,w}}\right) \\ =&1 - \prod_{i=1}^{\kappa}\left(1 - \frac{\sum_{w \in S_i^{(t)} \setminus S_i^{(t-1)}} \frac{1}{\rho}}{1 - \sum_{w \in S_i^{(t-1)}} \frac{1}{\rho}}\right) \\ =&1 - \prod_{i=1}^{\kappa}\left(1 - \frac{|S_i^{(t)} \setminus S_i^{(t-1)}|}{\rho - |S_i^{(t-1)}|}\right) \end{aligned}$$

Since the in-degree of every node is same, we get the same probability distribution over the active sets in both the cases. □

Consequently we obtain the result that the expected number of active nodes in both the **TA** and **NA** cases are the same for the networks with constant in-degree. In the general case when the networks do not have a constant degree for every node but the randomization over the network structure preserves a fixed degree distribution (as in the case of Power Law or Spatially Clustered networks) we may obtain similar results. However the probability that a node becomes active in time step $t+1$, given that it was not active till time step $t$ would be calculated for a node $v$ with $\kappa(v) = \kappa \neq 0$ and degree $d \neq 0$. Assuming that the distribution over the values $d$ would be the same at the time step $t$ in both the cases (notice that the distribution over the values $\kappa$ would be the same for both the cases since the initial distribution of node resources are the same), we will obtain similar results. Our experimental results show that this observations about the Sticky model carries over to the general model. In all of the simulation experiments we observe that the estimations of the expected values of the different metrics (total participation, total adoption, resource utilization etc.) for both the **TA** and **NA** cases are almost identical.
